# Laser-Induced Erasable and Re-Writable Waveguides within Silver Phosphate Glasses

**DOI:** 10.3390/ma15092983

**Published:** 2022-04-20

**Authors:** Konstantinos Tsimvrakidis, Ioannis Konidakis, Emmanuel Stratakis

**Affiliations:** Institute of Electronic Structure and Laser (IESL), Foundation for Research and Technology-Hellas (FORTH), 71110 Heraklion, Greece; stratak@iesl.forth.gr

**Keywords:** silver phosphate glass, ultrafast laser processing, erasable waveguides, direct laser writing, photonics

## Abstract

Femtosecond direct laser writing is a well-established and robust technique for the fabrication of photonic structures. Herein, we report on the fabrication of buried waveguides in AgPO_3_ silver metaphosphate glasses, as well as, on the erase and re-writing of those structures, by means of a single femtosecond laser source. Based on the fabrication procedure, the developed waveguides can be erased and readily re-inscribed upon further femtosecond irradiation under controlled conditions. Namely, for the initial waveguide writing the employed laser irradiation power was 2 J/cm^2^ with a scanning speed of 5 mm/s and a repetition rate of 200 kHz. Upon enhancing the power to 16 J/cm^2^ while keeping constant the scanning speed and reducing the repetition rate to 25 kHz, the so formed patterns were readily erased. Then, upon using a laser power of 2 J/cm^2^ with a scanning speed of 1 mm/s and a repetition rate of 200 kHz the waveguide patterns were re-written inside the glass. Scanning electron microscopy (SEM) images at the cross-section of the processed glasses, combined with spatial Raman analysis revealed that the developed write/erase/re-write cycle, does not cause any structural modification to the phosphate network, rendering the fabrication process feasible for reversible optoelectronic applications. Namely, it is proposed that this non-ablative phenomenon lies on the local relaxation of the glass network caused by the heat deposited upon pulsed laser irradiation. The resulted waveguide patterns Our findings pave the way towards new photonic applications involving infinite cycles of write/erase/re-write processes without the need of intermediate steps of typical thermal annealing treatments.

## 1. Introduction

The interaction between laser and matter has been studied over the past decades offering an increasingly wide range of scientific and technological applications. More specifically, the direct laser writing (DLW) technique and the use of ultrashort laser sources in transparent glasses is of great interest as it offers 3D structuring inside bulk transparent materials [1]. It also poses an additional advantage in terms of simplicity when compared to other lithography inscription techniques that require more steps as for instance photomask, etch, and deposition processes, while are limited to 2D structuring [1,2,3,4]. As for instance Fernandez at al. reported on the development of optical waveguide amplifiers in phospho-tellurite glasses by means of fs laser irradiation [2]. Meanwhile ultrafast laser-matter interactions on transparent materials have studied extensively to reveal the damage thresholds and breakdown points of laser processing [3,5]. As a result, numerous studies demonstrate the feasibility of the fs lasers for the development of advanced photonic circuits and waveguide platforms within oxide glasses [6,7,8,9].

It is well reported that focusing of femtosecond laser pulses (typically in the near-IR) of appropriate intensity inside the glass volume can result in permanent modification of the glass structure [10]. This is due to photo-induced change of the refractive index (Δn) attributed to nonlinear absorption effects [11,12]. Depending on the laser parameters used, such refractive index changes are classified into three main types, namely type I, II and III [13]. Type I modifications consist of smooth and permanent isotropic changes. In this case the respective refractive index changes are intrinsic modifications of the glass matrix. Namely, they are typically induced due to the local heating and melting which is followed by a rapid cooling phase. This process causes a variation in the glass density, due to ion exchange or, the formation of color centers within glass [9,10,13,14,15,16,17,18]. Type II changes appear due to a strong linear birefringence (i.e., in the order 10^−2^). Such changes arise by the creation of a local plasma giving rise to the formation of nanostructures and self-organized nanogratings [19,20]. Lastly, type III modifications are consisted by the formation of voids within glasses [21], and within crystalline materials [22]. Such voids are caused by an excessive plasma production and successive Coulomb explosions [21,22]. Recently, a new type was demonstrated in silver containing glasses coined as type A (Argentum). Type A refractive index changes occur due to the formation of fluorescent silver clusters that act as an extrinsic agent. Unlike the other glass modification types, type A changes require much lower laser fluence and repetition rate of the order of MHz [23,24]. In fact, silver containing glasses offer an attractive platform for the fabrication of advanced 3D waveguides by means of fs laser irradiation [25,26,27].

Notably, research studies on direct laser writing of waveguides into glassy materials are dominated by silica-based compounds, which is favored by its excellent transparency [13]. However, among all glasses, pure silica exhibits the highest transition and melting temperatures, usually exceeding the 1000 °C [28]. On the other hand, silver metaphosphate glass (AgPO_3_) is a soft glass with much lower transition temperature (T_g_) of ~190 °C. The selection of AgPO_3_ glass as a matrix for waveguides inscription can be also favored by its capability of hosting high concentrations of rare earth ions that make it ideal for lasing and amplifier applications operating in the C-band telecom wavelengths [7,29,30]. In this work, we demonstrate the inscription of optical buried waveguides through femtosecond direct laser writing within AgPO_3_ glasses. Remarkably, the employment of this silver-rich ‘soft’ phosphate glass enables the erasing and re-writing of the waveguide features by means of the same femtosecond laser source and wavelength, upon altering the irradiation conditions. Notably, waveguide erase has been reported before through high temperature thermal annealing treatments [31]. However, to the best of our knowledge, the presented all-laser write/erase/re-write process of this study is the first demonstration of in-situ erasing and re-writing of waveguides in metaphosphate glasses, induced by photothermal processes, under the same wavelength, without the need of conventional thermal annealing.

## 2. Materials and Methods

**Figure 1:**
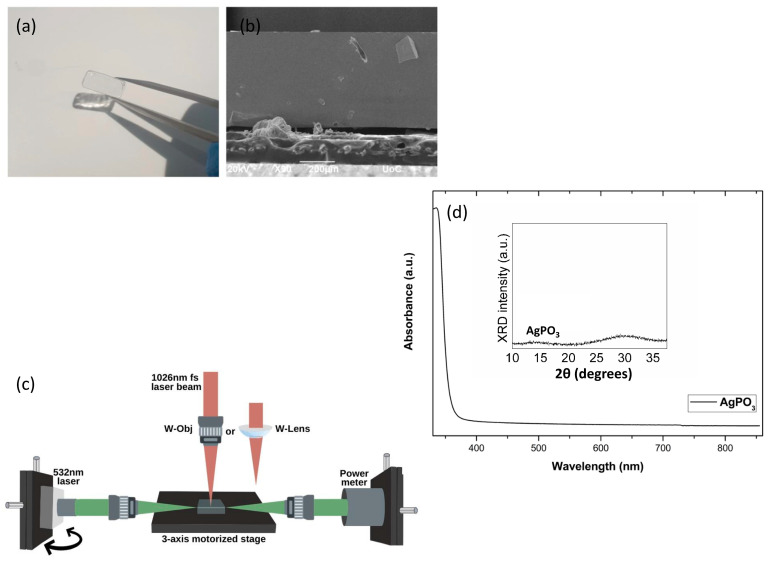
(**a**) Photograph of the as-prepared silver metaphosphate AgPO_3_ glass sample; (**b**) SEM image of the as-prepared AgPO_3_ glass cross-section (top-view). (**c**) Schematic representation of the experimental apparatus. (**d**) Typical optical absorbance profile and XRD pattern of AgPO_3_ glass.

The silver metaphosphate glass, AgPO_3_, was prepared by weighing and mixing equimolar high-purity AgNO_3_ (99.995%) and NH_4_H_2_PO_4_ (99.999%) dry powders into a platinum crucible, following a standard procedure [32,33]. The crucible was then transferred to an electrical furnace with an initial temperature of 180 °C and steadily heated up to 300 °C allowing the controlled removal of the volatile gas products. The temperature was then increased to 350 °C and was kept steady for a few minutes. The melt was then rapidly poured into a custom-made mold while pressed with a silicon wafer stamp to produce AgPO_3_ glasses of rectangular shapes with around 1 cm length, 0.5 cm width and thickness of 0.3 cm, as shown in Figure 1a. The employment of the silicon wafer during melt-quenching ensures the formation of a smooth upper surface suitable for laser processing. No further polishing was performed, while the samples were stored under vacuum. A scanning electron microscopy (SEM) image of the as-prepared phosphate glass cross-section is also shown in Figure 1b. Inspection of Figure 1b reveals a smooth surface and a glass interior without the presence of agglomerated silver particles.

The experimental setup for the direct laser inscription of the waveguides is shown in Figure 1c. The laser source used was an Yb: KGW femtosecond laser (Pharos femtosecond) from Light Conversion, with a maximum pulse energy of 1.5 mJ and a maximum laser power of 1.5 W at 1 kHz repetition rate. The fs laser was producing polarized optical pulses of 170 fs, center emission wavelength at 1026 nm and tunable repetition rate up to 200 kHz. The laser beam pulses were focused on the phosphate glass sample using either a plano-convex lens or a 10× Mitutoyo Plan apochromatic infinity corrected objective lens. The plano-convex lens was able to create a Gaussian ~60 μm diameter beam spot at 200 mm focal length, and the objective lens at 20 mm focal length resulted in a much smaller in diameter ~10 μm beam spot. Both spots were measured by a CMOS camera at 1/e^2^ within the Rayleigh range of the focal plane. To assist in distinguishing the two pathways employed each time throughout the text, we will use the terms W-Lens for the plano-convex lens, and W-Obj for the Mitutoyo ×10 objective lens. Table 1 summarizes the employed laser irradiation parameters for the waveguide fabrication, erase, and re-write processes.

As depicted schematically in Figure 1c the glass substrates were mounted on a motorized three-axis stage. Following the DLW process, the phosphate glass facets were cut to reveal the fabricated waveguides. Visualization of the fabricated waveguides was accomplished with the help of a 532 nm continuous wave laser source. The visible laser beam was focused by a different 10× objective lens mounted on a three-axis stage and used to probe light in the waveguide through different incident angles. The collection of the waveguided light was made by an identical objective lens, also mounted on a three-axis stage and was driven into a sensor. The DLW process was performed in room temperature under ambient conditions.

In order to explore the structural integrity of the metaphosphate glass samples subjected to the DLW process, Raman spectroscopy was used with acquired spectra from either the surface or the cross-section of the processed areas. Raman spectroscopy was carried out after each processing step to ensure that no significant structural modifications were made to the glass network. For sample excitation a 532 nm laser was employed. Finally, the optical absorbance and the X-ray diffraction spectra of the glass waveguides were studied to provide further evidence that the optical properties and the amorphous nature of the glass have not changed upon the described femtosecond laser process when compared to the pristine AgPO_3_, as shown in Figure 1d.

## 3. Results and Discussion

For the fabrication of waveguides, the developed phosphate glass samples were irradiated with femtosecond laser pulses and the laser beam was focused through the plano-convex or the objective lens inside the body of the glass (Figure 1c), forming optical waveguides a few hundreds of micrometers below the surface (~200 µm).

**Figure 2:**
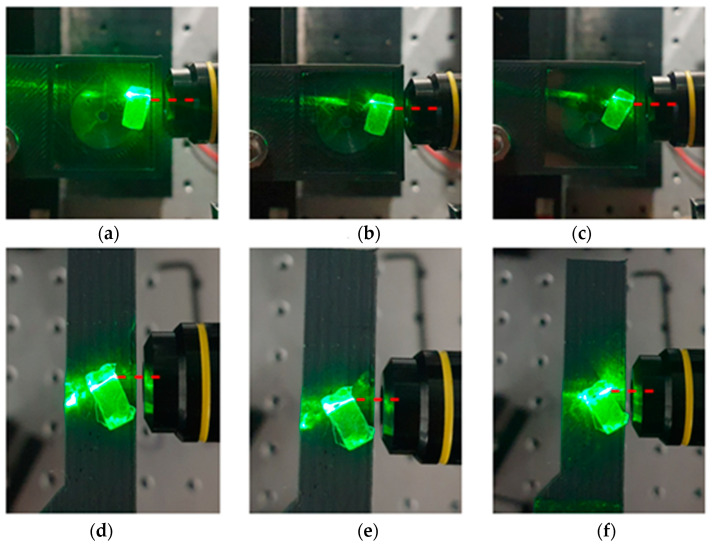
Phosphate glass waveguide (W-Lens) with plano-convex lens (**a**–**c**) positioned at (**a**) 15°, (**b**) 20° and (**c**) 25° of the incident visible light, and (W-Obj) with objective lens (**d**–**f**) at (**d**) 30°, (**e**) 35° and (**f**) 40°, showing the threshold of the acceptance angle. Red dashed lines indicate the zero-degree incident probe light. Writing irradiance for both W-Lens and W-Obj glasses was set to 200 kHz repetition rate, 3 μJ pulse energy and 5 mm s^−1^ scanning speed.

**Figure 3:**
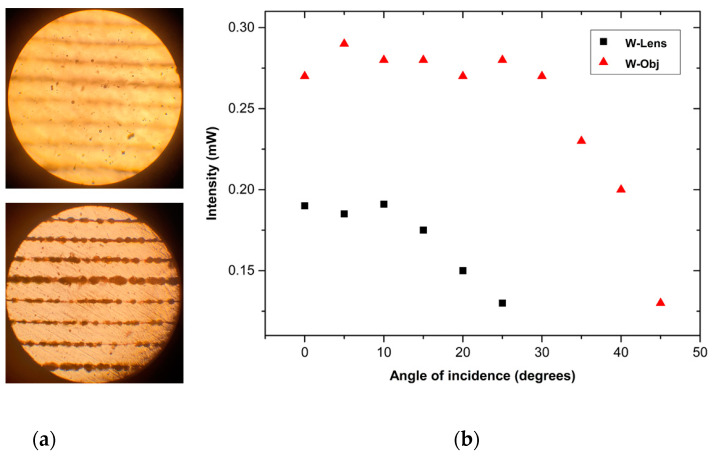
(**a**) Optical microscope image of a W-Obj processed phosphate glass focused on its surface (top) and 300 μm below the surface (bottom). For scale illustration purposes please consider SEM photos. (**b**) Intensity of the transmitted light versus incident angle.

The optimal pulse energy range for the formation of the waveguides was found to be in the range 1–3 μJ, while the acceptable translation speed levels were found to be between 1 and 5 mm s^−1^. Figure 2a–c present indicative snapshots of a fabricated waveguide upon employing the W-Lens approach, while Figure 2d–f depict the corresponding ones for the W-Obj, in both cases formed with a pulse energy of 3 µJ (equal to a fluence of 3 J cm^−2^) and 5 mm s^−1^ scanning speed. In order to account for the acceptance angle threshold, the fabricated waveguide was positioned at various angles with respect to the incident visible green laser beam. A schematic representation of the process of probing visible light into the waveguide was shown in Figure 1c. By employing the objective lens over the plano-convex lens the femtosecond laser beam was focused on a smaller spot with a much lower Rayleigh range, leading to a more efficient energy accumulation on the targeted area within the glass. This can be seen in Figure 3a, where optical microscopy images of waveguides fabricated with the objective lens (W-Obj) are clearly seen by focusing below the surface while back illuminating with white light. These photos reveal that the laser modification of the glass occurs within the bulk, rather than on the surface. In terms of light transmission, the critical angle is found to be about 25° for the W-Lens waveguides, while that for the W-Obj ones was found to be considerably higher up to 40°, resulting to numerical aperture (NA) values close to 0.4 and 0.6, respectively. Figure 3b presents the optical power transmitted through the W-Obj and W-Lens waveguides as a function of the angle between the glass plane and the incident probe light. It is observed that the optical power of light travelling through the waveguide is not affected until the critical angle is reached, above which the transmitted light significantly decreases. The optical losses of both W-Lens and W-Obj waveguides were calculated and listed in Table 2. Namely, for W-Obj the average transmittance is about 61% and drops down to 28% for the wider incident angle of 45°, whereas for the W-Lens it starts from an average of 40% down to 27% for the incident angle of 25°. Notably, when the power meter is removed from the waveguide facet it reveals zero light transmission, thus, proving that the transmitted light is due to laser-induced confined waveguide features, rather than typical light scattering.

**Figure 4:**
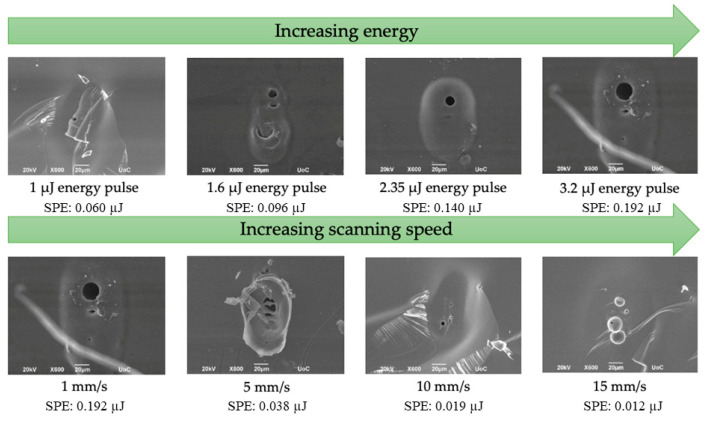
SEM images from the cross-section of processed phosphate glasses. Top line shows the effect of increasing optical energy at a constant scanning speed of 1 mm s^−1^, while the bottom line depicts the effect of the increasing inscription scanning speed keeping constant the energy of the incident laser pulses at 3.2 μJ. The specific point energy (SPE) values are also included.

The aforementioned processing parameters for optimum waveguide inscription, were determined via a thorough parametric study upon varying the energy pulse and laser scanning speed. Figure 4 presents SEM images of the waveguides cross-section upon increasing laser energy while maintaining constant scanning speed of 1 mm s^−1^, and upon increasing scanning speed at a stable energy pulse of 3.2 μJ. At the constant scanning speed of 1 mm s^−1^, inspection of Figure 4 reveals that a circular void channel starts to form in the phosphate glass interior when the laser power raises above 1.6 μJ. Indeed, the so-formed voids in the interior of the phosphate glass are increasing in size as the applied optical pulses increase in energy. On the other hand, it is found that when the energy pulse is kept stable at 3.2 μJ, the observed channel appears to be more profound (larger void created) for the slower scanning speed of 1 mm s^−1^. Indicatively, the diameter of the void channel can reach values of around 20 μm, for the processing conditions of 3.2 μJ and 1 mm s^−1^. In particular, keeping constant the pulse energy at 3.2 μJ while increasing the scanning speed, and thus the number of the effective laser pulses (N_eff_ = [beam spot diameter × repetition rate]/scanning speed), results to less pronounced modifications within the glass as the scanning speed increases, i.e., smaller voids created.

**Figure 5:**
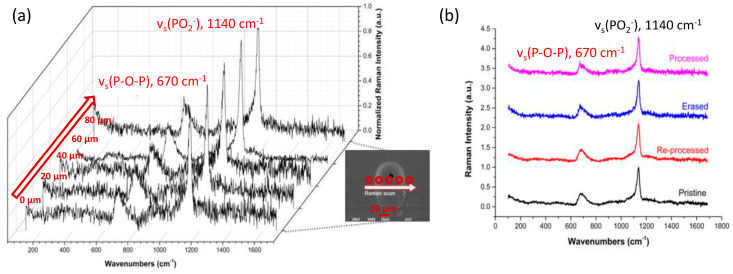
(**a**) Raman mapping spectra of a processed AgPO_3_ glass cross-section with steps of ~20 μm starting right before the processed area on pristine glass. Inset SEM image shows the processed area scanned and the direction of the Raman scanning across the processed area and the laser-induced void (for clarity the arrow and dots are shown below the void). (**b**) Surface Raman spectra of pristine, processed, erased and reprocessed AgPO_3_ glass. All spectra were normalized at 1140 cm^−1^ to facilitate comparison.

Raman spectroscopy was employed on the surface and the cross-sectioned processed area in order to investigate any possible structural modifications of the phosphate glass network upon laser processing and the resulted voids formation. Figure 5a presents Raman spectra from different locations of the cross-section of a W-Lens waveguide inscribed with an energy pulse of 2.35 µJ (fluence of 2.35 J cm^−2^) and 1 mm s^−1^ scanning speed. All Raman spectra of Figure 5a are dominated by two main bands at around 1140 cm^−1^ and 670 cm^−1^. The former is attributed to the symmetric stretching vibration of terminal PO_2_ group v_s_(PO_2_^−^). The Raman profile at around 670 cm^−1^ originates from the symmetric stretching movement of the P-O-P bridges v_s_(P-O-P) within the phosphate backbone [34,35]. The relative intensities of the two Raman features provide direct evidence on the population of terminal and bridging phosphate entities within the network. For the purposes of comparison, all spectra of Figure 5a are normalized at the v_s_(PO_2_^−^) band at 1140 cm^−1^. It becomes apparent that the relative intensities remain similar across the laser-processed region (inset of Figure 5a), and similar to those of the pristine glass (Figure 5b). This provides strong evidence that the connectivity of the phosphate network remains largely unaffected by the DLW process. Remarkably, these findings are in agreement with a previous study where erasable laser induced periodic surface structures were formed on the surface of the AgPO_3_ glass by means of femtosecond laser processing [35].

**Figure 6:**
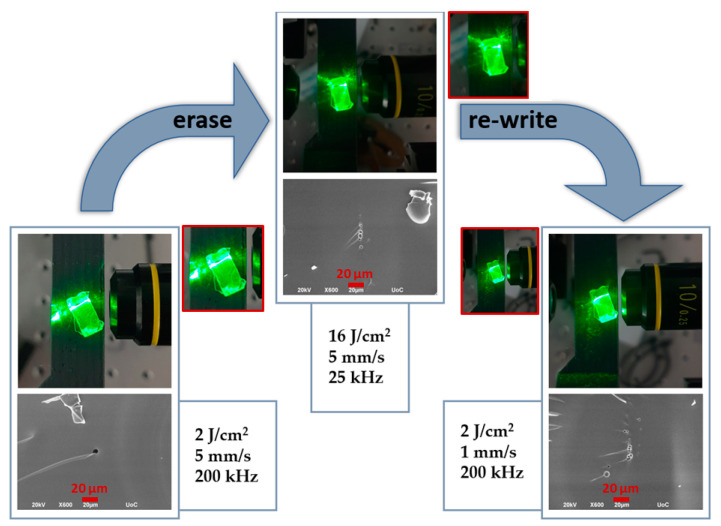
Images visualizing the waveguiding effect along with the corresponding SEM images at the cross-section of the processed glass areas. After initial fabrication (**left**), at the erase of the written waveguide (**middle**) and the re-make of the waveguide (**right**) at the same glass spot. The insets (red squares) show enlarged photos of the waveguides.

More importantly, the so formed waveguides can be erased and re-written upon sequential scanning with the same laser source, while slightly altering the irradiation conditions. As an example, the waveguide in Figure 6 is formed at 2 J/cm^2^ and 5 mm s^−1^ scanning velocity, with 200 kHz repetition rate, leading to Neff = 1000 pulses. This waveguide was erased upon using a fluence of 16 J/cm^2^, but lowering the repetition rate down to 25 kHz, i.e., for Neff = 50 pulses. Remarkably enough, the waveguide was inscribed for a second time upon irradiation with parameters close to the ones used for the initial writing process. More specifically, the repetition rate was set again at 200 kHz, while the energy fluence used was 2 J/cm^2^, with a scanning speed of 1 mm s^−1^, i.e., for Neff = 2000 pulses. Worth noticing is the fact that for the reforming of the waveguide an increase in the Neff was necessary.

Raman spectroscopy is again employed to investigate any potential modifications of the phosphate glass network during the writing-erase-re-writing process. As shown in Figure 5b, the relative intensities of the two main bands for (vs(PO^2−^) and vs(P-O-P)) entities remain almost identical with no notable shifts on their positions. Moreover, all spectra are in good agreement with the one of the pristine AgPO_3_ glass, also shown in Figure 5b. This confirms that the write/erase/re-write processing cycle causes no structural modifications to the phosphate glass network. Namely, the connectivity of the phosphate backbone remains unaffected without any changes to the relative populations of terminal (non-bridging) and bridging oxygen atoms [35]. Additionally, the optical absorbance profiles and X-ray diffraction (XRD) patterns of the processed samples were found to be identical to these of the as-prepared AgPO_3_ glasses shown in Figure 1d. These similarities indicate that the phosphate glass’s optical properties have not changed when compared to the corresponding data of pristine AgPO_3_ glass [33,35], while no crystallization has occurred due to laser processing for waveguide inscription.

Following the revealed phosphate network structural integrity, amorphic nature, and unchanged optical absorption profile upon laser processing, we now consider the photothermal effects on the AgPO_3_ glass upon the waveguide inscription, erasing, and re-write stages. Notably, the direct DLW energy fluence and scanning speed conditions applied in this study for the initial waveguide inscription are within the range of previously reported values for the processing of AgPO_3_ glass [35]. As highlighted in one of our previous studies, the soft nature of AgPO_3_ glass (T_g_ 190 °C), allows an extensive experimentation with these processing parameters. Based on this, herein the parameters were optimized for achieving feasibly the writing, erase, and re-write waveguiding process, without causing any structural modifications to the phosphate network.

At the first step of the cycle enough photothermal energy is transferred to the glass network for the formation of the channel void. Namely, as depicted in Figure 6 left, initially a mild irradiation (2 J cm^−2^, 5 mm s^−1^, 200 kHz) was able to create a waveguide channel void in the glass several micrometers below the surface, evidence of a Type III waveguide. Next, upon applying the appropriate erasing parameters (16 J cm^−2^, 5 mm s^−1^, 25 kHz) the waveguiding effect is eliminated, and the void presented before is replaced with multiple smaller ones in a larger area that do not suffice for effective light transmission through the glass body (Figure 6 middle). In particular, upon increasing the energy fluence while decreasing dramatically the laser’s repetition rate down to 25 kHz the effective pulses applied are of much higher energy while the number of these pulses per spot are notably lower. Consequently, the void initially created below the glass’s surface, is modified and/or removed, and the waveguiding means are destroyed. Finally, by re-illuminating the erased area with the previously mentioned parameters (2 J cm^−2^, 1 mm s^−1^, 200 kHz), multiple channel voids of slightly larger diameter are created, proven to be sufficient for waveguiding as the probe light is once again guided from one facet to the other (Figure 6, right). Thus, the nature of the aforementioned photothermal processes is believed to be entirely non-ablative. Rather differently, it becomes apparent that the removal of the initial writing lies on the local relaxation of the glass network as induced by the applied local heat transfer during further laser irradiation caused by the accumulated laser pulses and the additional photothermal effects. In principle, by deleting the structures formed and basically ‘restoring’ the glass to its previous state makes room for de novo processing of the already processed area. Based on the above findings, the demonstrated write/erase/re-write cycle could be readily repeated multiple times, rendering the developed waveguide fabrication route suitable for a wide variety of photonic applications and optical memory components. Finally, since the waveguide features are known to be dependent on the phosphate glass components, apart from the processing parameters, it is noted that tuning the glass composition alongside the DLW parameters should provide an additional tool for the further development of phosphate glass-based waveguides with advanced reversible features.

## 4. Conclusions

In conclusion, we have demonstrated the laser induced writing, erase and re-inscription of buried waveguides in metaphosphate glasses via employing a single femtosecond laser source. The erased waveguides could be re-written by further irradiating the processed glass area under similar, to the initial inscription, writing parameters. In particular, for the initial waveguide writing the employed laser irradiation power was 2 J/cm^2^ with a scanning speed of 5 mm/s and a repetition rate of 200 kHz. Upon enhancing the power to 16 J/cm^2^ while keeping constant the scanning speed and reducing the repetition rate to 25 kHz, the so formed patterns were readily erased. Then, upon using a laser power of 2 J/cm^2^ with a scanning speed of 1 mm/s and a repetition rate of 200 kHz the waveguide patterns were re-written inside the glass. The fabricated waveguides exhibited losses between 1.91 and 5.39 dBm depending on the incident angle of the light source. The whole process was shown to be non-ablative, lying only on the local relaxation of the glass network induced by photo-thermal processes. This enables performing arbitrary number of write/erase/re-write cycles. Our work highlights the perks of the soft nature of the phosphate glass family and will potentially enable new photonic applications, especially in situations where a material structure is required to be removed and re-formed via an all-laser based process, such as in advanced waveguides and optical memory applications.

## Figures and Tables

**Table 1:** Summary of employed laser irradiation conditions for the waveguide fabrication, erase, and re-write processes.

	Waveguide Fabrication	Waveguide Erasure	Waveguide Re-Writing
Energy fluence	2 J/cm^2^	16 J/cm^2^	2 J/cm^2^
Repetition rate	200 kHz	25 kHz	200 kHz
Translation speed	5 mm/s	5 mm/s	1 mm/s

**Table 2:** Optical losses of W-lens and W-Obj fabricated waveguides.

	W-Lens		W-Obj	
Angle of Incident (Degrees)	Transmittance (%)	Loss (dBm)	Transmittance (%)	Loss (dBm)
0	41	3.74	61	2.22
5	40	3.98	63	1.91
10	42	3.63	62	2.06
15	39	4.1	62	2.06
20	33	4.77	61	2.14
25	27	5.56	62	2.06
30	-	-	61	2.14
35	-	-	50	2.91
40	-	-	44	3.52
45	-	-	28	5.39

## Data Availability

Not applicable.
